# First characterization of the venom of the endemic coral snake *Micrurus camilae* (Serpentes: Elapidae) from Colombia: Proteome, toxic activities, immunorecognition, and neutralization by antivenoms

**DOI:** 10.1371/journal.pntd.0013941

**Published:** 2026-02-17

**Authors:** Jeisson Gómez-Robles, Paola Rey-Suárez, Julián Fernández, Mónica Saldarriaga-Córdoba, Mahmood Sasa, Bruno Lomonte, Vitelbina Núñez

**Affiliations:** 1 Grupo de Investigación en Toxinología, Alternativas Terapéuticas y Alimentarias (TOXATA). Universidad de Antioquia, Medellín, Colombia; 2 Facultad de Ciencias Exactas y Aplicadas, Institución Universitaria ITM, Medellín, Colombia; 3 Instituto Clodomiro Picado, Facultad de Microbiología, Universidad de Costa Rica, San José, Costa Rica; 4 Escuela de Medicina Veterinaria, Centro de Investigación en Recursos Naturales y Sostenibles (CIRENYS), Universidad Bernardo O'Higgins, Santiago, Chile; 5 Museo de Zoología, Centro de Investigaciones en Biodiversidad y Ecología Tropical, Universidad de Costa Rica, San José, Costa Rica; 6 Escuela de Microbiología, Universidad de Antioquia, Medellín, Colombia; Instituto Butantan, BRAZIL

## Abstract

Thirty-one species of *Micrurus* (coral snakes) are distributed in Colombia. However, functional and proteomic analyses of their venoms have only been performed on six of them. *Micrurus camilae* is endemic to Colombia, and no information exists regarding its venom. The proteome of *M. camilae* venom, its biochemical and toxic activities, immunorecognition, and neutralization by commercial equine antivenoms and an experimental one prepared in rabbits are here reported. In addition, the phylogenetic position of *M. camilae* within the genus was explored. The venom was characterized by RP-HPLC, SDS-PAGE, and nESI-MS/MS, and functional analyses were performed using *in vitro* (proteolytic, coagulant, phospholipase A_2_, and L-amino acid oxidase activity) and *in vivo* (myotoxic, edematogenic, hemorrhagic) assays. Immunorecognition and neutralization were evaluated using ELISA and mouse lethality, respectively. To determine phylogenetic relationships, sequences of the mitochondrial *ND4* gene from 48 *Micrurus* species were analyzed. The venom proteome revealed a PLA_2_-rich phenotype and identified 17 protein families, the four most abundant being PLA_2_, LAO, 3FTx, and MP. The myotoxic and hemorrhagic activities observed in mice correlated with the relative abundance of PLA_2_s and MPs, respectively. Furthermore, the i.p. lethal effect in mice was associated with only one fraction, a 3FTx. Two commercial equine antivenoms (INS-anticoral and ICP-anticoral) immunologically recognized both the whole venom and the chromatographic fractions by ELISA. However, they did not neutralize venom lethality in mice in a preincubation assay. On the other hand, the experimental rabbit antivenom was shown to recognize the whole venom and its fractions and, although it did not completely neutralize lethality, it prolonged mouse survival by several hours compared to the venom-only control. Our phylogenetic hypothesis showed *M. camilae* within the mipartitus group as a sister species of *M. mipartitus*.

## Author summary

Snakebite envenomation is a global public health problem. Snakes of the genus *Micrurus* are distributed throughout the Americas, and due to the composition of their venoms, they induce a neurotoxic syndrome that can be fatal if antivenom is not administered. Colombia harbors 31 *Micrurus* species, among which *M. camilae* is endemic, and its venom has not been previously studied. In this work, we present the first characterization of the composition and biological activities of *M. camilae* venom. In addition, we evaluated whether commercial antivenom and an experimental antivenom could neutralize its toxicity.The results revealed that the venom is composed of 17 protein families, with phospholipases A₂ being the most abundant, followed by L-amino acid oxidases, three-finger toxins, and metalloproteinases. The venom exhibited lethal activity, which was attributable to a single fraction corresponding to a three-finger toxin. Moreover, the venom showed myotoxic, edematogenic, and hemorrhagic effects, the latter being uncommon among venoms of this genus. The commercial antivenom did not neutralize the lethal effect of the venom; however, a species-specific experimental antivenom was able to prolong survival compared with the control. These findings highlight the urgent need to advance venomic and antivenomic studies on clinically relevant *Micrurus* species in Colombia.

## Introduction

Snakebite envenomation is a significant public health issue in many tropical and subtropical regions [1]. Globally, an estimated 5 million snakebites occur each year, resulting in 1.8–2.7 million envenomations and 80,000–138,000 deaths. In addition, amputations and other permanent disabilities are frequent outcomes of this neglected disease [1,2]. Beyond the physical harm, snakebites have also been increasingly recognized for their psychological and socioeconomic consequences. Mental health disorders, emotional trauma, and loss of productivity are well-documented among survivors, particularly in low-resource settings, [3–7]. Most snakebite cases occur in Africa, Asia, and Latin America, disproportionately affecting impoverished rural communities in low-income countries, where access to healthcare is limited and health systems are under-resourced [1,2,8–11]. Most of the clinically significant envenomations are caused by snakes of the Viperidae and Elapidae families. Within Elapidae, the genus *Micrurus* (New World coral snakes) comprises 83 recognized species, distributed from southern United States to northern Argentina [12]. In Colombia, approximately 6,200 snakebite cases are reported annually [13], of which 1–2% are attributed to *Micrurus* species [14–17].

Antivenoms remain the only effective specific treatment for snakebite envenomation [1,18]. The development of improved antivenoms, optimization of therapeutic protocols, and exploration of novel therapeutic alternatives depend on a robust scientific foundation and comprehensive knowledge of venomous snakes and their venoms. In recent years, an increasing knowledge has been built through proteomic analyses, identification of clinically relevant venom components, and epidemiological surveillance of snakebite incidence, among other approaches [2,9,19].

To date, the venom proteomes of 23 *Micrurus* species have been characterized, representing less than one-third of this genus diversity. These venoms most typically contain proteins from seven to nine families, although the number may range from three to sixteen. These studies have revealed evolutionary trends in venom composition, including a notable dichotomy in the relative abundance of the two major protein families that are consistently present: phospholipases A_2_ (PLA_2_s) and three-finger toxins (3FTxs) [20,21].

Colombia hosts 31 species of *Micrurus* [12] but functional and proteomic venom analyses have only been conducted for six of them: *M. mipartitus* [22], *M. dumerilii* [23], *M. medemi* and *M. sangilensis* [24]*, M. helleri* (previously named *M. lemniscatus helleri*; [24,25] and *M. nigrocinctus* [26]*.* Similarly, phylogenetic relationships among Colombian coral snakes remain poorly understood and have only been explored in a few species, including *M. dissoleucus* [27]*, M. helleri* [28], *M. dumerilii* [29] and *M. nigrocinctus* [26]. Likewise, the pre-clinical evaluation of commercial antivenoms against the venoms of species found in Colombia has been limited to a few cases [30–32].

**Fig 1:**
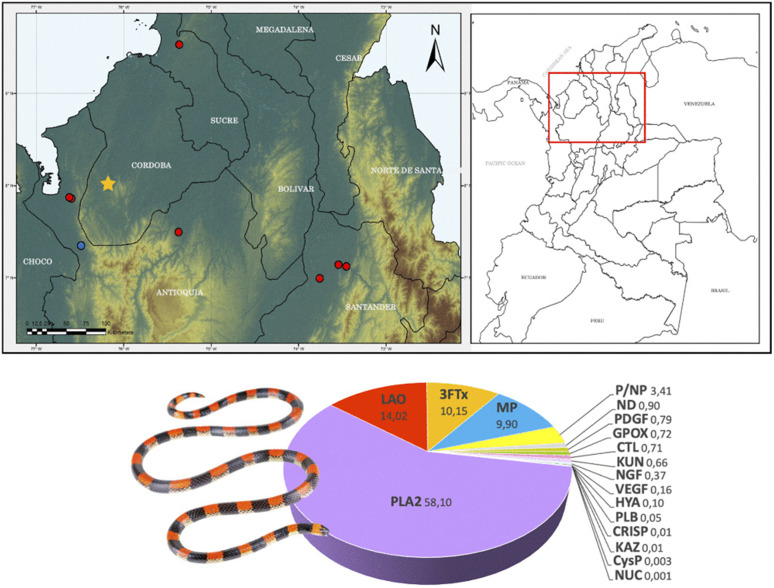
Geographic distribution of *Micrurus camilae* in Colombia and proteomic composition of its venom. **(A)** Map showing the known distribution of *M. camilae* based on published records. The inset highlights the region where the species has been reported. The type locality is indicated by a star, the origin of the specimen analyzed in this study is marked with a red dot, and other reported records from the literature are shown with blue dots [34–36,59]. Photograph by Jose Vieira, ExSitu Project. **(B)** Proteomic composition of *M. camilae* venom (full details of identifications are provided in S1 Table). The pie chart shows the relative abundance (%) of venom components classified into protein families, including phospholipase A_2_ (**PLA**_**2**_), L-amino acid oxidase (**LAO**), three-finger toxins (**3FTx**), metalloproteinases (**MP**), peptides/non-proteinaceous components (**P/NP**), nucleotidases (**ND**), platelet-derived growth factors (**PDGF**), glutathione peroxidase (**GPO**X), C-type lectins (**CTL**), Kunitz-type inhibitors (**KUN**), nerve growth factor (**NGF**), vascular endothelial growth factor (**VEGF**), hyaluronidase (**HYA**), phospholipase B (**PLB**), cysteine-rich secretory proteins (**CRISP**), Kazal-type inhibitors (**KAZ**), cysteine protease inhibitors (**CySP**), and nucleases (**NUC**).

*Micrurus camilae* is a coral snake endemic to Colombia, characterized by a distinctive pattern of black and pale-yellow rings, the latter dorsally interrupted by a red stripe — an identifying feature among other coral snakes [33]. This species is distributed across the lowland inter-Andean regions of the departments of Córdoba, Antioquia, Sucre, César, and Santander [33–36] (Fig 1). In some areas, its distribution overlaps with human habitats, posing a potential risk for snakebites. However, most of the available data on this species pertains solely to its geographical distribution. Therefore, this study aims to characterize the venom proteome of *M. camilae* and its biochemical and toxic activities, as well as to evaluate its immunorecognition and neutralization by two commercially available equine antivenoms and an experimental rabbit antivenom.

## Materials and methods

### Ethics statement

All procedures were performed in accordance with the guidelines License No. 160 of 2024 and No. 166 of 2025 issued by the Institutional Committee for the Care and Use of Laboratory Animals (CICUA) from the University of Antioquia.

### Venoms and antivenoms

Venom was manually extracted from a single adult female *M. camilae* collected in the Urabá region, Department of Antioquia, Colombia (Fig 1). The specimen was maintained in captivity at the institutional serpentarium of the University of Antioquia. All procedures were conducted under the Ministry of Environment genetic resource access permit RGE: 0156-15.

Two commercially available equine antivenoms: INS-anticoral (produced by the Instituto Nacional de Salud, Colombia, using the venoms of *M. dumerilii, M. mipartitus, M. isozonus* and *M. surinamensis* as immunogens; batch No. 23AMP01, expiry date: November 2025); and ICP-anticoral (CoRal-ICP) (produced by Instituto Clodomiro Picado, University of Costa Rica, using the venom of *M. nigrocinctus* as immunogen; batch No. 7040723ACLQ, expiry date: July 2026) were used for the immunochemical and neutralization assays. The antivenoms were tested before their expiration dates.

### Molecular analysis

A buccal swab sample was obtained from the *M. camilae* specimen and DNA extraction was performed using E.Z.N.A. Omega Tissue DNA Kit (Cat. D3396-01), following the manufacturer’s protocols. Gene amplification was performed by polymerase chain reaction (PCR) using the following primers for *ND4* gene (*ND4*F: 5’ CACCTATGACTACCAAAAGCTCATGTAGAAGC-3´ [37]; LEU: 5´-ATTACTTTTACTTGGATTTGCACCA-3´ [38]. PCR reactions were set up to a final volume of 25 µL, using 1 µL genomic DNA (2 ng/µL), 0.5 µL of each primer (0.2 µM), 2.5 µL of 10X PCR buffer (1X), 0.5 µL total dNTPs (0.2 mM), 0.75 µL of MgCl_2_ (1.5 mM), 0.1 µL of Platinum Taq DNA Polymerase (1 U), and 19.15 µL of H_2_O. Typical amplification conditions involved initial denaturation at 94 ºC for 5 min, followed by 35 cycles with a denaturation step at 95 ºC for 45 s, an annealing stage at 55 ºC for 45 s, an extension at 72 ºC for one min and a final extension at 72 ºC for 10 min. Amplicons were separated by electrophoresis on 1.5% agarose gels in 0.5x TBE buffer, dyed with GelRed Nucleic Acid Gel Stain (Biotium, Inc.) and visualized under UV light. We performed Sanger sequencing in an automated capillary ABI3500 sequencer (Applied Biosystems), at the AUSTRAL omics (Santiago, Chile). The DNA sequences were edited (Trim Ends and De Novo Assemble) and aligned in Geneious Prime v2025.0.3 [39]. For *ND4* and *cyt-b* genes, the nucleotide sequences were translated into proteins to evaluate the reading frame and ensure the absence of premature stop codons or other nonsense mutations in GeneDoc [40]. Novel sequence was deposited in GeneBank (accession number PX021565).

### Phylogenetic reconstruction

A total of 48 coral snake sequences were included in the phylogenetic analysis, with *Calliophis bivirgatus* and *Micruroides euryxanthus* designated as outgroups. The protein-coding *ND4* gene was aligned using the MACSE algorithm [41], applying the vertebrate mitochondrial genetic code in PhyloSuite v1.2.3 [42]. Since most taxa available for reconstructing the phylogenetic position of *M. camilae* from Colombia included *ND4* sequences, phylogenetic inference was conducted based on this gene. Maximum Likelihood (ML) analyses were conducted using IQ-TREE v1.6.8 [43], with substitution model selection performed using ModelFinder [44] under the Bayesian Information Criterion (BIC). Node support was assessed using 10,000 ultrafast bootstrap replicates. Bayesian Inference (BI) analyses were conducted using MrBayes v3.2.7 [45]. The GTR + I + G substitution model (nst = 6, rates = invgamma) was specified, with a flat Dirichlet prior for base frequencies (statefreqpr = dirichlet(1,1,1,1)) and variable rate priors across partitions (ratepr = variable). All substitution parameters were unlinked across partitions, although only a single partition was used in this analysis. Two independent MCMC runs with four chains each were executed for 5 million generations, sampling every 1,000 generations. A burn-in of 25% (1,250 samples) was applied prior to summarizing the posterior distribution. Tree topologies were summarized using a majority-rule consensus tree with the Halfcompat option. Posterior probabilities were calculated from the remaining trees.

Convergence was assessed by examining the effective sample size (ESS) of all parameters using Tracer v1.5 [46], with all ESS values exceeding 300, indicating adequate sampling. The final phylogenetic tree was visualized and edited using iTOL [47].

### Chromatographic and electrophoretic profiles

Two mg of *M. camilae* venom were dissolved in 200 µL of 0.1% trifluoroacetic acid (solution A), centrifuged at 1250 × g for 5 min, and then fractionated on a C_18_ column (250 × 4.6 mm, 5 µm particle size) using an Agilent 1200 HPLC system, with monitoring at 215 nm. Elution was performed at a flow rate of 1 mL/min using the following gradient toward solution B (acetonitrile containing 0.1% TFA): 5% B for 5 min, 5–15% B over 10 min, 15–45% B over 60 min, and 45–70% B over 12 min [48]. Peaks were manually collected, dried in a vacuum centrifuge, redissolved in water and analyzed by SDS-PAGE under non-reducing conditions. Twenty µg of each fraction were separated on a 15% gel using a Mini-Protean Tetra Cell electrophoresis system (Bio-Rad, Hercules, CA, USA) at 150 V. Molecular weight markers (Precision Plus Protein Standards, Broad Range, Bio-Rad, Hercules, CA, USA) were used, and proteins were visualized using Coomassie Brilliant Blue R-250 staining.

### Proteomic profiling of *M. camilae* venom

The electrophoretic bands from each chromatographic fraction were excised from the gel and reduced with dithiothreitol and alkylated with iodoacetamide, followed by in-gel digestion with sequencing-grade trypsin in an automated processor (Intavis, Germany) according to the manufacturer’s instructions. The resulting tryptic peptides were analyzed by nESI-MS/MS using a nano-Easy 1200 chromatograph in-line with a Q-Exactive Plus mass spectrometer (Thermo Fisher).

Five μL of each tryptic digest were loaded on a C_18_ trap column (75 μm × 2 cm, 3 μm particle size), washed with 0.1% formic acid (solution A), and separated at a flow rate of 200 nL/min through a C_18_ Easy-spray column (15 cm × 75 μm, 3 μm particle size). Elution was carried out using a gradient towards solution B (80% acetonitrile, 0.1% formic acid) over 45 min (1–5% B in 1 min, 5–25% B in 30 min, 25–79% B in 6 min, 79–99% B in 2 min, 99% B for 6 min). The mass spectra were acquired in positive mode at 1.9 kV, capillary temperature of 200 ˚C, using 1 μscan in the range 400–1600 m/z, maximum injection time of 100 ms, AGC target of 3 × 10^6^, and a resolution of 70,000. The top 10 ions with 2–5 positive charges were fragmented with an AGC target of 1 × 10^5^, a maximum injection time of 110 ms, a dynamic exclusion time of 5 s, and a resolution of 17,500. The resulting MS/MS spectra were processed against protein sequences contained in the UniProt/SwissProt Serpentes database (https://www.uniprot.org) using PEAKS X (Bioinformatics Solutions) and matches were assigned to known protein families by similarity. Cysteine carbamidomethylation was set as a fixed modification, while deamidation of asparagine or glutamine, and methionine oxidation were set as variable modifications, allowing up to 3 missed cleavages by trypsin. Parameters for match acceptance were set to FDR < 0.1%, detection of at least one unique peptide, and -10lgP protein score ≥50. The relative abundances (expressed as percentage of the total venom proteins) of the different protein families were calculated as the ratio of the sum of the areas of the reverse-phase chromatographic peaks containing proteins from the same family to the total area of venom protein peaks in the reverse-phase chromatogram. For reverse-phase fractions containing several protein bands in SDS-PAGE, their proportions were assessed by densitometry, using ImageLab v2.01 (Bio-Rad). When several proteins were detected in the same SDS-PAGE band, their proportions were estimated on the basis of the total intensity of matching tryptic peptides in MS/MS analysis. Finally, protein family abundances were estimated as the percentages of the total venom proteome.

### Monoisotopic mass determination of a lethal toxin

A Q-Exactive Plus mass spectrometer (Thermo Fisher) with a heated electrospray ionization (HESI) ion source was used to determine the monoisotopic mass of a lethal toxin separated in peak 10 by RP-HPLC. The toxin was dissolved in 50% acetonitrile and 0.1% formic acid and analyzed by direct infusion (flow rate 5 μL/min). MS spectra were acquired in positive mode, using 3.9 kV spray voltage, full MS scan range from 800 to 2500 m/z, 140000 resolution, and an AGC target of 3 × 10 ^6^). The monoisotopic molecular mass was calculated by deconvolution of the isotope-resolved multiply charged MS1 mass spectra.

### Amino acid sequencing of a lethal toxin

The complete amino acid sequence of a lethal toxin present in peak 10 was determined by tandem mass spectrometry analysis of its tryptic peptides. Using the same methodology as described above, tryptic peptides were *de novo* sequenced with assistance from PEAKS X (Bioinformatics Solutions). The theoretical monoisotopic mass of the sequence was compared and confirmed with the experimental monoisotopic mass obtained in the previous section. The 3D structure of toxin was modelled using AlphaFold3 [49]. The sequence of lethal toxin was compared with other three-finger toxins of *Micrurus* by multiple alignment using Geneious Prime version 2025.1.2.

### Enzymatic and toxic activities of *M. camilae* venom

The venom PLA_2_ activity was evaluated using the chromogenic substrate 4-nitro-3-octanoyloxy-benzoic acid (4-NOBA), following the protocol by [50]. In brief, 5 μg of venom were dissolved in 25 μL of buffer (10 mM Tris-HCl, 10 mM CaCl_2_, 100 mM NaCl, pH 8.0) and mixed with 25 μL of 4-NOBA (1 mg/mL in acetonitrile) and 200 μL of the same buffer. After incubation for 60 min at 37°C, absorbance was measured at 405 nm using a microplate reader.

Coagulant activity was evaluated following the method described by [51]. Fifty μg of venom, in 100 μL phosphate-buffered saline; PBS: 0.12 M NaCl, 40 mM phosphate buffer, pH 7.2) was added to 200 μL of citrated human plasma, pre-incubated at 37°C, and clotting time was recorded.

L-amino acid oxidase (LAO) activity was tested using the protocol described by [52]. Five μg of venom (diluted in 10 μL of water) were added to 90 μL of a reaction mixture containing 250 μM L-Leucine**,** 2 mM *o*-phenylenediamine, and 0.8 U/mL horseradish peroxidase, in 50 mM Tris-HCl buffer (pH 8.0)**.** After incubation at 37°C for 60 min, the reaction was stopped with 50 μL of 2 M H_2_SO_4_**,** and absorbance was recorded at 492 nm**.**

Proteolytic activity was assessed using azocasein as a substrate, according to [53]. Twenty μg of venom (in 20 μL of buffer: 25 mM Tris-HCl, 0.15 M NaCl, 5 mM CaCl_2_, pH 7.4) were added to 100 μL of azocasein (10 mg/mL in the same buffer), and incubated at 37°C for 90 min**.** The reaction was terminated by adding 200 μL of 5% trichloroacetic acid**.** After centrifugation, 100 μL of the supernatant were mixed with 100 μL of 0.5 M NaOH**,** and absorbance was measured at 450 nm.

In the above *in vitro* assays, the venoms of *M. dumerilii* and *M. mipartitus* were included for comparison, under the same conditions. All samples were assayed in triplicates.

For *in vivo* assays, male and female Swiss Webster mice weighing 18–20 g was used. All procedures were performed in accordance with the guidelines License No. 160 of 2024 and No. 166 of 2025 issued by the Institutional Committee for the Care and Use of Laboratory Animals (CICUA) from the University of Antioquia.

Edema-forming activity was evaluated following the method of [54]. Groups of three mice (18–20 g body weight) received 5 μg of venom dissolved in 50 μL of PBS, injected subcutaneously into the right hind footpad. The contralateral footpad received PBS only, as a control. After 2 hr**,** mice were euthanized via CO_2_ inhalation and the footpads were dissected and weighed, to determine their differences.

Myotoxic activity was assessed by intramuscular (i.m.) injection of 5 μg of venom (in 50 μL PBS) into the gastrocnemius muscle of groups of three mice (18–20 g body weight). Control animals received PBS only. After 3 hr, blood was collected from the tail, and plasma creatine kinase (CK) activity was measured using an UV kinetic assay (Weiner Lab, CK-NAC UV-AA) [55].

Hemorrhagic activity was tested following the method described by [56]. A group of three mice (18–20 g body weight) were injected intradermally (i.d.) with 50 μg of venom in 100 μL of PBS. After 2 hr**,** the animals were euthanized by CO₂ inhalation, and the inner surface of the skin was dissected and examined to measure the area of the hemorrhagic lesion.

Finally, the lethal activity of the venom was evaluated by intraperitoneal (i.p.) injection in groups of three mice (18–20 g body weight). Mice received different doses of venom, dissolved in 300 μL PBS, and mortality was assessed after a 48-hour period, to estimate median lethal dose (LD_50_) by probit analysis [57]. In addition, each of the major RP-HPLC venom fractions (2, 10, 11, 13, 19, 27, 29, 30, 32, 33, 35, 39, 40) was screened for lethal activity by i.p. injection of 50 μg, dissolved in 250 μL PBS.

### Rabbit experimental antivenom production

One adult New Zealand rabbit was immunized with *M. camilae* venom for six months, using a scheme that started with 400 μg of venom followed by four additional boosters (790 μg, 1180μg, 1770 μg, and 1170 μg). Along the process, and one week after the last booster, blood samples were obtained, and sera were stored at -20°C. The serum IgG fraction was obtained by precipitation of non-Ig proteins with caprylic acid as described by [58]. Aliquots of caprylic acid (Sigma, Saint Louis, MO, USA) were gradually added to the serum until they reached 5% of the total volume. After centrifugation, the supernatant was dialyzed using cellulose membranes (3500 Mw cut-off; Fisherbrand) against PBS, and then against distilled water. Finally, this IgG fraction was lyophilized and stored at -20°C. For all assays a stock of 30 mg/mL of total IgG was used (Experimental antivenom).

### Immunorecognition and neutralization by antivenoms

Antibody titration curves of the INS-anticoral and ICP-anticoral antivenoms were performed against whole *M. camilae* venom using an enzyme-linked immunosorbent assay (ELISA). Each well of a microplate was coated with 0.1 μg of complete venom diluted in 100 μL of coating buffer (0.1 M Tris, 0.15 M NaCl, pH 9.0) and incubated overnight at room temperature. The wells were then blocked with 100 μL of 1% bovine serum albumin in PBS (BSA-PBS; 0.04 M phosphate, 0.12 M NaCl, pH 7.2) for 90 min. Serial dilutions of the antivenoms (from 1:500–1:100,000), or a non-immune equine serum as negative control, were added to the wells and incubated for 90 min at room temperature. After washing, an anti-horse or rabbit IgG antibody conjugated with horseradish peroxidase (1:8000; Sigma-Aldrich) was added and incubated for an additional 90 min. Following a final wash step, 100 μL of the substrate solution (2 mg/mL *o*-phenylendiamine in 0.1 M sodium citrate buffer, pH 5.0, supplemented with 4 μL of 30% H₂O₂ per 10 mL of final solution) was added to each well. Absorbance was measured at 490 nm using a Multiskan Sky microplate reader (Thermo Scientific, Waltham, MA, USA). Experimental antivenom against *M. camilae* was evaluated in the same conditions, using as second rabbit IgG antibody conjugated with horseradish peroxidase (1:8000; Sigma-Aldrich) and a non-immune rabbit serum as negative control.

A second experiment was conducted to evaluate the antigenic recognition of the INS-Anticoral or experimental antivenom against the major venom fractions obtained by RP-HPLC. To this end, the plate was coated with 0.1 μg/well of each fraction and ELISA was performed as described above using a 1:1000 dilution of the antivenom.

Finally, the ability of the INS-anticoral and experimental antivenom against *M. camilae* to neutralize the lethal effect of *M. camilae* venom was evaluated by a pre-incubation assay**.** Groups of three mice (18–20 g body weight) were injected i.p. with 500 μL of a solution containing 94 μg of *M. camilae* venom (equivalent to 2 × LD_50_), previously incubated for 30 min at 37°C with the antivenom at a ratio of 0.2 mg of venom per mL of antivenom. This venom/antivenom proportion was selected on the basis of previous neutralization studies performed with other *Micrurus* venoms [30]. A control group received the same venom dose, pre-incubated with PBS instead of antivenom. Deaths were recorded at 48 hours.

## Results

**Fig 2:**
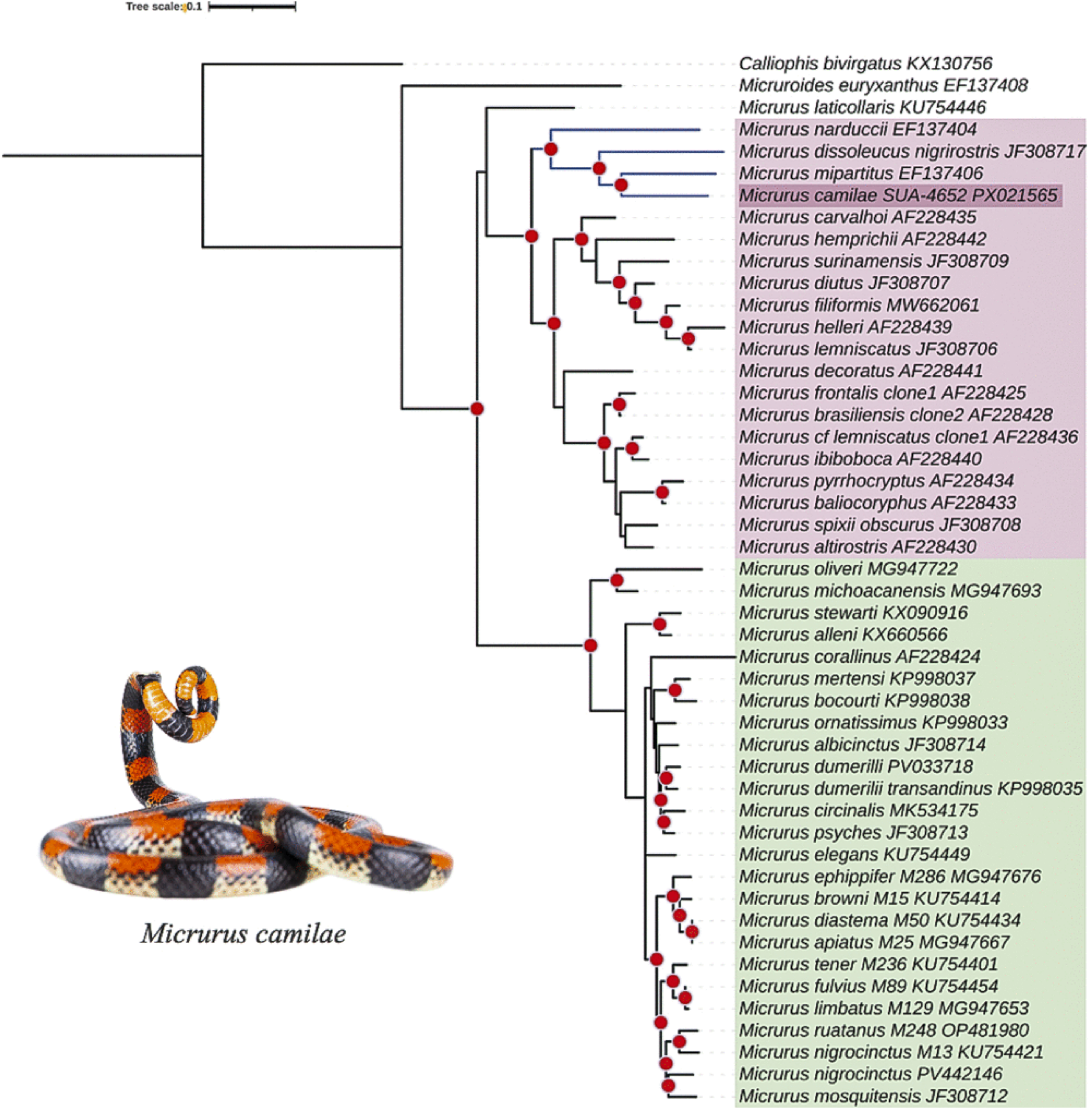
Phylogenetic relationships of *Micrurus* species inferred from mitochondrial *ND4* sequences. Phylogenetic tree inferred using the Maximum Likelihood (ML) method in IQ-TREE, based on a 627 bp alignment of the mitochondrial *ND4* gene. The GTR + G + I substitution model was selected as the best-fit model by the software. Node values represent ultrafast bootstrap support (UFBoot) based on 10,000 replicates. *Calliophis bivirgatus* and *Micruroides euryxanthus* were included as outgroups. The triadal clade is highlighted in pink, and the monadal clade in green. Nodes with bootstrap support >80% and Bayesian posterior probability >0.95 are marked with red circles. Although ML and Bayesian Inference (BI) yielded congruent topologies, only the ML tree is presented. Photograph represent the usual behavior of this species of curling its tail, in defense situations. Photo: Jose Vieira, ExSitu Project.

The phylogenetic relationship of *M. camilae* to other *Micrurus* species was inferred from the comparison of their mitochondrial *ND4* gene sequences. The recovered phylogenetic tree showed two main well-supported monophyletic clades, Monadal and Triadal group (boostrap support [bs] = 83/100). Within the latter, *M. camilae* forms a group with *M. narduccii*, *M. dissoleucus*, and *M. mipartitus*, showing a closer relationship with the latter (bs = 90) (Fig 2).

**Fig 3:**
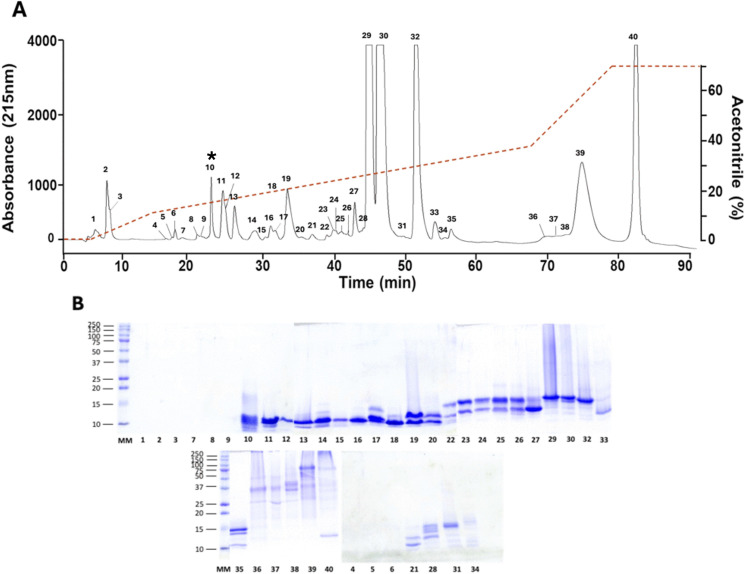
Separation and analysis of *M. camilae* venom proteins by RP-HPLC and SDS-PAGE. **(A)** Reverse-phase high-performance liquid chromatography (RP-HPLC) profile of *M. camilae* venom. Two mg of crude venom were applied to a C_18_ column (4.6 × 250 mm) and eluted with an acetonitrile gradient (dotted line). The asterisk indicates the only lethal fraction of venom, by i.p. injection of mice at a dose of 50 μg. **(B)** SDS-PAGE (15%) of the collected HPLC fractions, under reducing conditions. Molecular mass markers are indicated at the left, in kDa.

The RP-HPLC profile of *M. camilae* venom showed 40 fractions (Fig 3A). Each of these was analyzed by SDS-PAGE. The first nine chromatographic peaks showed no staining, suggesting they could correspond to very small peptides or to non-proteinaceous components. Most fractions showed two to three protein bands in the range of 10–20 kDa. Only the fractions corresponding to the last eluting region of the chromatogram (36 and 40) presented bands above 20 kDa (25 and 150 kDa, respectively) (Fig 3B). The proteomic analysis of fractions identified components in three molecular mass ranges: high molecular mass components (>20 kDa), components with molecular masses between 13 and 20 kDa, mainly represented by PLA_2_s, and low molecular mass components (6–10 kDa), mainly corresponding to 3FTxs (details summarized in S1 Table).

The *M. camilae* venom proteome comprises at least 17 protein families. PLA_2_s were the most abundant component in the venom, representing 58%, followed by LAOs (14%), 3FTxs (10%), and MPs (9.9%). Components from families such as ND, PDGF, GPOX, CTL, KUN, NGF, VEGF, HYA, PLB, CRISP, KAZ, and CysP were found in smaller proportions (<1%). Additionally, 3.41% (P/NP) of the components corresponded to either small peptides or non-protein components (fractions 1–9) (Fig 1; S1 Table).

**Fig 4:**
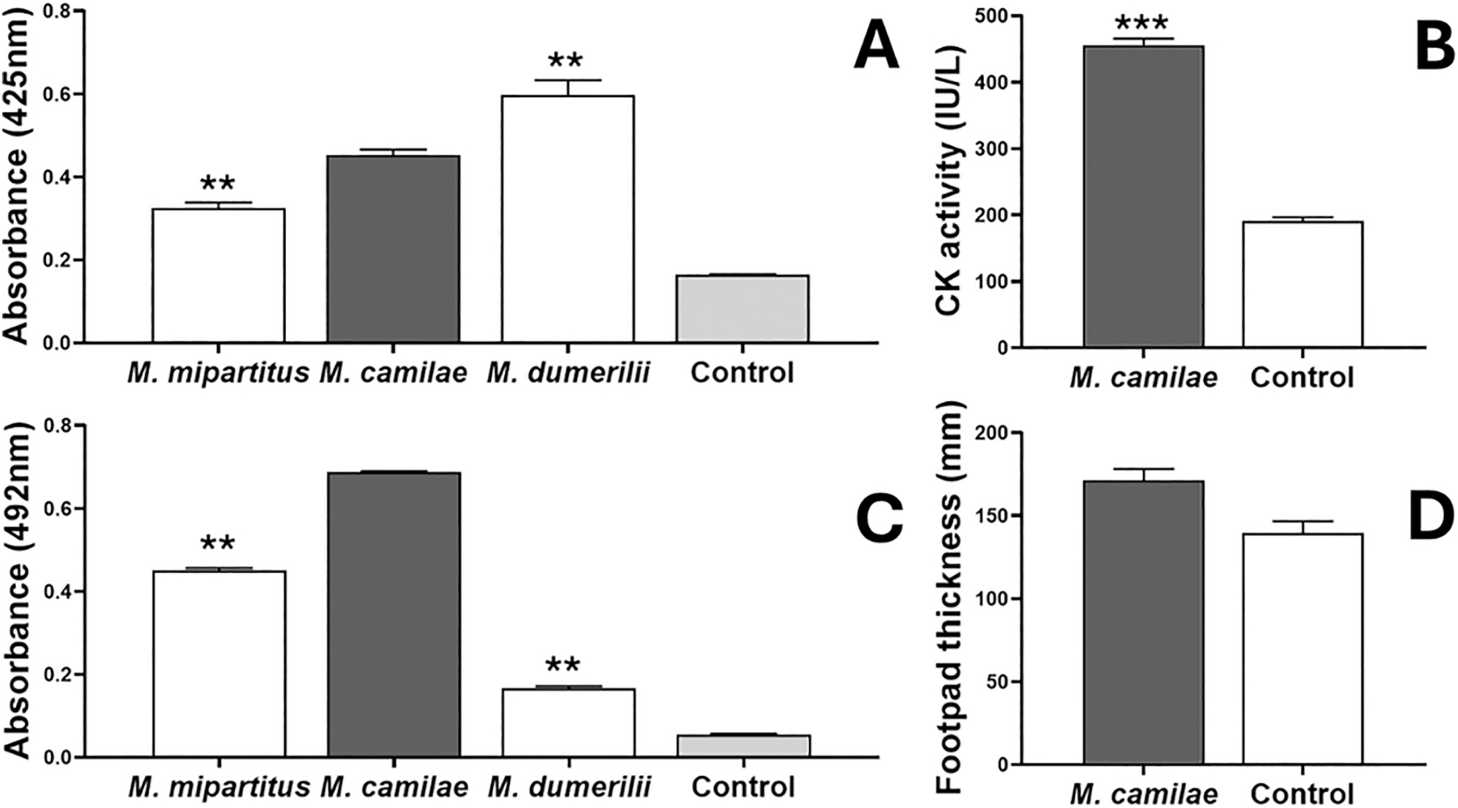
Biochemical and toxic activities of the whole venom of *Micrurus camilae.* **(A)** Phospholipase A_2_ (PLA_2_) activity on 4-nitro-3-octanoyloxy-benzoic acid, using 5 μg of venom. **(B)** Myotoxic activity induced by intramuscular injection of venom (5 μg) in groups of three mice; phosphate-buffered saline (PBS) was used as a control. **(C)** L-amino acid oxidase (LAO) activity, using 20 μg of venom. **(D)** Edematogenic activity induced by subcutaneous injection of venom (5 μg) into the right footpad of mice (n = 3); PBS was injected as control in left footpad. The venoms of *M. mipartitus* and *M. dumerilii* were included for comparative purposes in the *in vitro* assays. Asterisks indicate statistically significant differences (*** p < 0.001 ** p < 0.01) between venoms. Bars represent mean ± standard deviation (SD) of three replicates.

**Fig 5:**
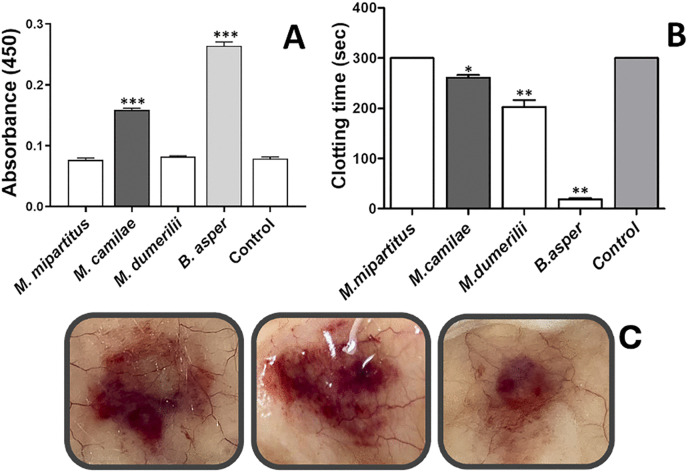
Biochemical and toxic activities of the whole venom of *M. camilae.* **(A)** Proteolytic activity, on azocasein, using 20 µg of venom **(B)** Coagulant activity on citrated human plasma using 50 µg of venom. **(C)** Hemorrhagic activity in mice using 50 µg of venom. The *in vitro* activities were compared with *M. mipartitus, M. dumerilii*, and *B. asper* venom (the latter included as a positive control). PBS was used as negative control. Statistically significant differences from controls are indicated by asterisks (*** p < 0.001 ** p < 0.01, * p < 0.05). Bars represent mean ± SD of three replicates.

*In vitro*, *M. camilae* venom showed PLA_2_ activity, which was significantly higher than that of *M. mipartitus* but lower than that of *M. dumerilii* (Fig 4A). On the other hand, the LAO activity of *M. camilae* venom was higher than that of both *M. mipartitus* and *M. dumerilii* venoms (Fig 4C). Similarly, the proteolytic activity of *M. camilae* venom was significantly higher than that observed for both venoms (Fig 5A). Finally, *M. camilae* venom showed a weak coagulant activity on citrated human plasma, lower than that recorded for *M. dumerilii* venom (Fig 5B).

*In vivo* experiments in mice showed that i.m. injection of *M. camilae* venom induced a significant increase in plasma CK activity, indicating its myotoxic activity (Fig 4B). This venom also induced a mild edema in the mouse footpad assay (23% increase in weight) although without reaching a statistically significant difference from controls (Fig 4C). Additionally, the venom induced a notable hemorrhagic effect (area = 133 mm^2^) when injected by intradermal route in mice (Fig 5C).

**Fig 6:**
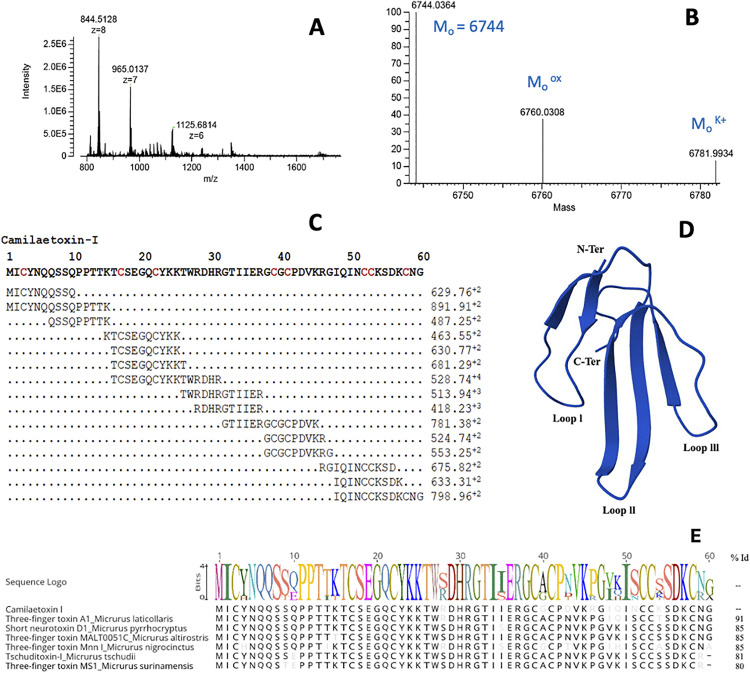
Characterization of the lethal fraction in *M. camilae* venom peak 10. Intact mass of Camilaetoxin-I. **(A)** Multicharge series; **(B)** Deconvolution, showing a monoisotopic mass of 6744 Da **(M**_**o**_), and additional peaks interpreted as an oxidized (+16, M_o_^ox^) form and a potassium (+38, M_o_^K+^) adduct; **(C)** Complete sequence of Camilaetoxin-I; **(D)** Three-dimensional structure modeled by AlphaFold3 **(E)** Alignment of Camilaetoxin-I with the 3FTx sequences with the highest identity; The colors in the sequence logo (consensus sequence) indicate the different amino acids.

Lethality analysis by i.p. injection of the whole venom estimated an LD_50_ of 46.7 μg for mice of 18–20 g (95% confidence limits: 39–81 μg) or 2.46 μg/g body weight. In addition, the i.p. lethality of the 14 most abundant peaks was evaluated individually, and only peak 10 showed toxicity at the tested dose of 50 μg/mouse (Fig 3A). This peak corresponded to a single protein identified as a 3FTx by MS/MS, with a monoisotopic mass of 6744 Da (Fig 6). Further tests with variable doses of this fraction estimated an i.p. LD_50_ of 3.2 µg/mouse (0.17 µg/g).

**Fig 7:**
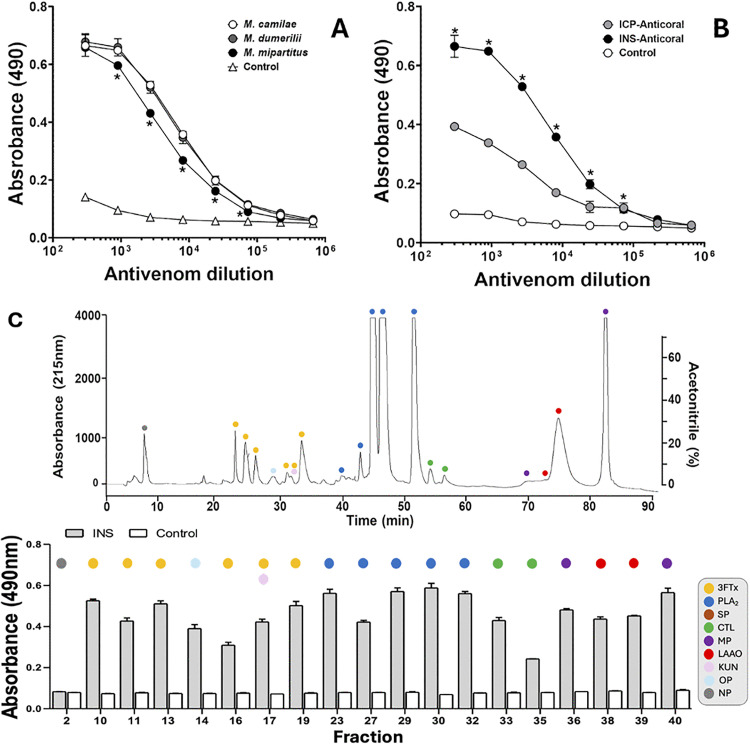
Immunorecognition of *Micrurus camilae* venom by commercial coral snake antivenoms. **(A)** Comparison of the immunobinding of the equine antivenom INS-Anticoral against the venoms of *M. camilae*, *M. mipartitus*, and *M. dumerilii*. **(B)** Comparative recognition of *M. camilae* venom by two antivenoms: INS-Anticoral and ICP-Anticoral. **(C)** Immunorecognition of the main RP-HPLC fractions (numbering as in the Fig 3) of *M. camilae* venom by the INS-Anticoral. Non-immune equine serum was used as a negative control. Asterisks indicate statistically significant differences (p < 0.05) between venoms **(A**) or respect to controls **(B and C)**. Each point represents mean ± standard deviation (SD) of triplicates.

The immunorecognition of whole *M. camilae* venom, as well as venoms of two medically important coral species in Colombia (*M. mipartitus* and *M. dumerilii*) by the INS antivenom was compared by ELISA. This antivenom showed significant immunorecognition for all three venoms tested, with similar titration curves for *M. camilae* and *M. dumerilii* venoms, and a slightly lower curve for *M. mipartitus* venom (Fig 7A). Nevertheless, when tested in a pre-incubation assay the INS antivenom did not neutralize the lethal effect of *M. camilae* venom in mice (Table 1). This result contrasts with the ELISA immunoprofiling of the antivenom, which showed the presence of antibodies able to bind most venom fractions, including the lethal F10 (Fig 7C).

**Table 1:** Neutralization of the lethal effect of *Micrurus camilae* venom in mice by INS-Anticoral and an experimental antivenom.

Group	Amount of venom (µg) 2 × LD_50_	Venom/ Antivenom (mg/mL)	Dead mice/Injected mice at 3 hours	Dead mice/Injected mice at 12 hours	Dead mice/Injected mice at 24 hours
***M. camilae* venom**	94	–	3/3	3/3	3/3
***M. camilae* venom + INS-Anticoral**	94	0.2/mL	3/3	3/3	3/3
***M. camilae* venom + experimental antivenom**	94	0.3/mL	0/3	0/3	3/3

In addition, the cross-recognition of *M. camilae* venom by the ICP-Anticoral against *M. nigrocinctus* was comparatively tested in parallel to the INS-Anticoral. Results showed a significant antibody binding by the ICP-Anticoral, but with a much lower signal than that obtained with the INS-Anticoral (Fig 7B). Therefore, venom neutralization by the ICP-Anticoral in mouse experiments was not attempted.

**Fig 8:**
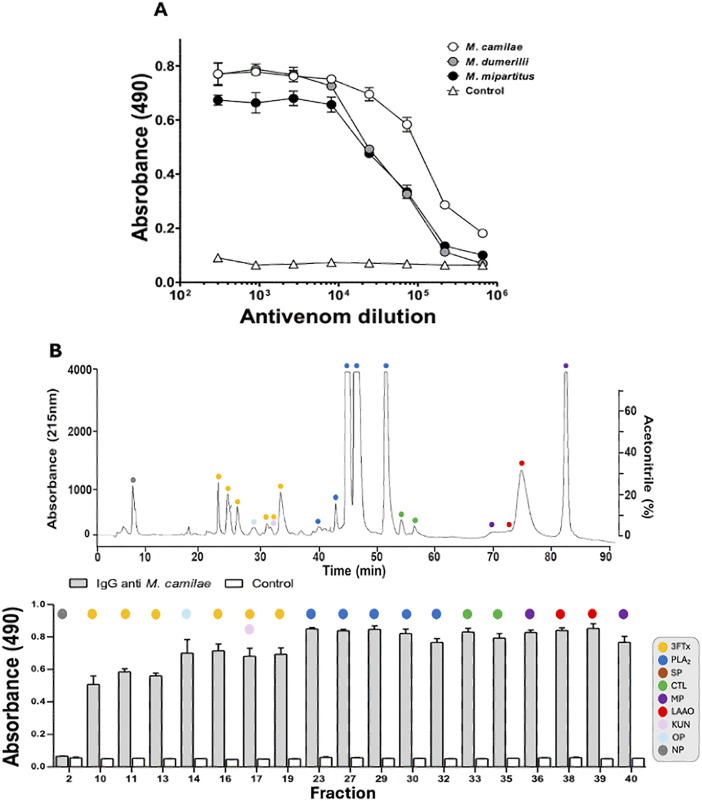
Immunorecognition of *Micrurus camilae* venom by experimental coral snake antivenom. **(A)** Comparison of the immunobinding of the experimental rabbit antivenom against the venoms of *M. camilae*, *M. mipartitus*, and *M. dumerilii*. **(B)** Immunorecognition of the main RP-HPLC fractions (numbering as in Fig 3) of *M. camilae* venom by an experimental antivenom. Non-immune rabbit serum was used as a negative control. Asterisks indicate statistically significant differences (p < 0.05) between venoms (**A**) or respect to controls **(B)**. Each point represents mean ± standard deviation (SD) of triplicates.

The immunorecognition of the rabbit experimental antivenom against whole venom of *M. camilae*, as well as of *M. mipartitus* and *M. dumerilii* was tested by ELISA. The antivenom showed significant recognition for all three venoms up to a dilution of 1:10,000. However, above this dilution, recognition was only maintained for *M. camilae* venom (Fig 8A). The ELISA immunoprofiling of its antivenom showed the presence of antibodies able to recognize most venom fractions, including the lethal F10 (Fig 8B). In a preincubation assay carried out in mice, the experimental antivenom prolonged survival, but did not neutralize the lethal effect of *M. camilae* venom at the end of the observation period (Table 1).

## Discussion

Snakebites inflicted by *Micrurus* species are considerably less frequent when compared to those caused by vipers [60]. Still, the neurotoxic actions of *Micrurus* venoms make these snakebites potentially lethal [17,61]. Furthermore, treatment for some species is limited due to the low cross-neutralization of their venoms by the available antivenoms [21]. In general, the study of coral snake venoms has been restricted by the difficulty in obtaining sufficient venom samples owing to low yields and poor survival of specimens in captivity [26,62].

In Colombia, the venoms of the most widely distributed and clinically important coral snake species have been studied. However, other species with more limited distribution remain largely undisclosed. One of them is *M. camilae*, an elusive species for which no information on its venom, or envenoming, is available. In this work, we were able to collect a single specimen and aimed to explore the venom characteristics as well as the phylogenetic relationships with other species of the genus. Results revealed that *M. camilae* groups within the *M. mipartitus* clade. Notably, however, the close phylogenetic relationship of *M. camilae* with *M. mipartitus* is in contrast with their venom compositions, as evaluated through proteomic analyses. While the venom of *M. camilae* showed a strong predominance of PLA_2_s (58%) over 3FTxs (10%), the venom of *M. mipartitus* has been previously shown to contain a predominance of 3FTxs (61%) over PLA_2_s (29%) [22]. In this regard, the venom composition of *M. camilae* fits within the group of PLA_2_-predominant venom phenotype proposed by Fernández et al. [63] for *Micrurus* species, together with the venoms of *M*. *dumerilii* [23], *M. helleri, M. medemi*, and *M. sangilensis* [24] from Colombia.

Interestingly, in *M. camilae* venom, the second most abundant toxin family was not the 3FTxs (10%), as is commonly observed in *Micrurus* species expressing a PLA_2_-rich phenotype [20]. Instead, L-amino acid oxidases (LAOs) accounted for 14% of the venom. Such abundance correlated with the higher LAO enzymatic activity compared to *M. mipartitus* and *M. dumerilii* venoms. Recent studies showed that in *M. sangilensis* venom LAOs were also abundant (9.17%; [24]), whereas very low proportions of this enzyme have been reported for some other *Micrurus* venoms such as *M. tschudii tschudii* (0.1%; [64]), *M. browni* (1.8%; [65]), or *M. ephippifer* [66]. The biological significance and overall roles in toxicity of LAO enzymes are poorly understood.

The presence of relatively abundant metalloproteinases in *M. camilae* venom (10%) resembles the proportions of this protein family described in the venoms of *M. helleri, M. medemi*, and *M. sangilensis* from Colombia (~13%, 10%, and 12%, respectively; [24]). Metalloproteinases are likely to be responsible for the hemorrhagic activity observed in *M. camilae* venom, an effect which is uncommon for *Micrurus* species. *M. averyi* venom has been reported to induce hemorrhagic activity in mice at a dose of 100 µg/mouse [67]**.** Additionally, the venom of *M. tener* (8 μg/mouse), administered intraperitoneally, was reported to induce moderate bleeding into the abdominal cavity and lungs [68]. In the present study, *M. camilae* venom induced hemorrhage after intradermal injection at a dose of 50 μg, an effect that could be related to its high proteolytic activity, as shown in the comparison with *M. dumerlii* and *M. mipartitus* venoms. This finding represents the first evidence of hemorrhagic action evoked by a *Micrurus* venom from Colombia by intradermal injection in mice. Further studies are needed to establish if there is a causal relationship of *M. camilae* venom metalloproteinases with hemorrhagic activity.

*M. camilae* venom showed a moderate myotoxic activity by intramuscular injection, likely caused by one or more of its PLA_2_s, in similarity with studies performed with several other *Micrurus* venoms [23,63,69–74]. The scarce amounts of venom available prevented performing more detailed functional analyses of the chromatographic fractions that would identify myotoxic components.

The venom of *M. camilae* was lethal by i.p. injection, with an estimated LD_50_ of 46.7 μg/mouse (2.5 μg/g). This lethal potency is lower in comparison to those previously reported for *M. dumerilii* (24 μg/mouse) [23] and *M. mipartitus* (9 μg/mouse) [75] venoms, under identical conditions. Unexpectedly, only one of the major venom peaks obtained by RP-HPLC showed i.p. lethal effect, at a screening dose of 50 μg. This peak was characterized as a 3FTx. None of the peaks corresponding to PLA_2_s caused lethality, in contrast to findings with other *Micrurus* venoms where some highly lethal PLA_2_s have been identified [23,70,76,77]. The possibility cannot be excluded that some PLA_2_s of *M. camilae* venom might be toxic to species other than mice, since rodents do not represent natural prey items for coral snakes. Other venom PLA_2_s of *Micrurus* species have also been found to lack lethality for mice [70,73,74]. On the other hand, some PLA_2_s have been reported to be lethal only when acting together with other venom components such as Kunitz-type peptides or 3FTxs [65,66,77,].

In this work, two equine antivenoms were evaluated for their ability to inmunorecognize *M. camilae* venom. The INS-anticoral antivenom showed much higher antibody titers against this venom, in comparison to the ICP-anticoral antivenom. However, unexpectedly, the INS antivenom did not neutralize the lethal effect of *M. camilae* venom in a pre-incubation assay, at a proportion of 0.2 mg of venom per mL of antivenom. Owing to limitations in injection volume (500 µL per mouse), we were unable to test higher proportions of antivenom to achieve lethality neutralization. However, since the antivenom was capable of immunorecognizing the whole venom and most of its fractions, the present results represent a further example reaffirming that immunorecognition does not necessarily imply that toxic effects such as lethality should be neutralized [30,78].

On the other hand, the experimental anti-*M. camilae* antivenom recognized the whole venom and its components. However, unlike commercial antivenoms, it prolonged survival time to 24 hours, suggesting that higher antibody titers and greater specificity (in comparison to antivenoms produced with other *Micrurus* venoms as immunogens) are required to develop antivenoms with improved neutralizing capacity.

The identification of toxins that play key roles in envenomation is a critical step toward improving the efficacy of current and next-generation antivenoms [79]. In this study, a lethal toxin from *M. camilae* venom was identified as a novel member of 3FTx family. This protein, which we here propose to name as Camilaetoxin-I, shares 85% and 84% sequence identity with a 3FTx of *M. nigrocinctus* from Costa Rica (UniProt:P805488.1; [80]) and Tschuditoxin-I (UniProt:4206391) of *M. tschudii tschudii* from the Peruvian Pacific coastal regions respectively [81]. Camilaetoxin-I was recognized by both commercial (equine) and experimental (rabbit) antivenoms, in contrast to Tschuditoxin-I, which was not recognized by the ICP-anticoral antivenom [81]. This finding suggests the presence of antigenic differences among *Micrurus* 3FTx toxins, even when they share high sequence identity.

Despite the low abundance of Camilaetoxin-I (1% of total venom proteins), this toxin exhibits potent lethality in mice, requiring only 3.2 µg/mouse to induce death. Its lethal potency is comparable to that reported for Mipartoxin-I from *M. mipartitus* venom [82]. These lethal 3FTx toxins represent promising candidates for the development of improved antivenom production strategies, particularly through their inclusion in immunizing mixtures.

## Conclusion

This first proteomic characterization of *M. camilae* venom, although limited by relying on a single specimen, revealed a PLA_2_-rich phenotype and identified 17 protein families in its composition. The four most abundant protein types corresponded, in descending order, to PLA_2_, LAO, 3FTx, and MP. Toxic activities such as myotoxicity and hemorrhagic effect appear to correlate with the relative abundances of PLA_2_ and MP enzymes, respectively, although causal relationships remain to be investigated. On the other hand, the i.p. lethal effect in mice might be associated only with a 3FTx fraction, Camilaetoxin-I. Two equine therapeutic antivenoms were able to immunorecognize the venom by ELISA, with the INS-anticoral resulting in a much stronger binding signal than the ICP-anticoral antivenom. The experimental antivenom against *M. camilae* immunorecognized most chromatographically-separated venom fractions, including the fraction having lethal effect and prolonged survival time by up to 24 hours, but did not neutralize venom lethality. A phylogenetic reconstruction of the mitochondrial *ND4* gene placed *M. camilae* as a sister species of the *M. mipartitis* group. Overall, this first study on *M. camilae* venom expands basic knowledge on the coral snakes of Colombia and their venoms. In addition, it provides relevant immunological information towards the goal of improving the species coverage of antivenoms intended for the treatment of envenoming by coral snakes in Colombia.

## Supporting information

S1 Table Assignments by nESI-MS/MS analysis of tryptic digests of protein bands excised from the SDS-PAGE separation of HPLC fractions of *Micrurus camilae* venom. Each column displays information related to: chromatographic peak or analyzed fraction (**Peak #**), initial representativeness of each protein or family in the total venom (**%**), normalized representativeness of each protein or family in the total venom (**%Final**), protein identifier in databases (**Accession**), statistical significance of the identification/score (**-10lgP**), sequence coverage achieved (**Cov (%)**), relative abundance based on the integrated ion area (**Area**), total number of assigned peptides (**#Pept**), number of unique peptides (**#Unique**), number of MS/MS spectra (**#Spectr**), average mass of the identified protein (**Avg. Mass**), related protein family (**Pr. family**), functional annotation of the protein and the reference species (**Description, Species**), unique peptides supporting the identification (**Supporting unique peptides**), monoisotopic mass (**Mass**), peptide length (**Length**), mass error (**ppm**), mass/charge ratio (**m/z**), and charge state (**z**) and post-translational modifications (**PTM**).
(XLSX)
